# The problem of weak AI-generated graphical abstracts

**DOI:** 10.17179/excli2026-9619

**Published:** 2026-06-19

**Authors:** Agapios Sachinidis, Wiebke Albrecht, Jan G. Hengstler

**Affiliations:** 1 Center for Physiology, Faculty of Medicine and University Hospital Cologne, University of Cologne, Robert-Koch-Str. 39, 50931 Cologne, Germany; 2 Department of Toxicology, Leibniz Research Centre for Working Environment and Human Factors (IfADo), Ardeystr. 67, Dortmund, 44139, Germany; 3 Department of Toxicology, Faculty of Chemistry, TU Dortmund, 44227 Dortmund, Germany

## ⁯⁯⁯⁯

In recent months, we have observed a growing number of manuscripts submitted with AI-generated graphical abstracts. At first glance, many of these figures appear highly professional. They are visually polished, colorful, and technically sophisticated. However, closer inspection often reveals a different picture: the scientific message is weak, unfocused, or partially lost behind decorative complexity.

A graphical abstract should not simply visualize an entire manuscript. Its purpose is to distill the central scientific insight into a clear and memorable visual message. This process requires intellectual selection and conceptual focus. In contrast, current generative AI systems often tend toward visual accumulation rather than scientific prioritization.

In our editorial experience, the problem is often not insufficient scientific quality of the manuscript itself. On the contrary, some manuscripts associated with weak graphical abstracts were scientifically excellent. In several cases, we had open and honest discussions with authors who explained that, after completing a demanding manuscript, a certain exhaustion had developed. AI tools created the impression that the final step - producing a graphical abstract - could easily be delegated and rapidly completed. Authors often entered large parts, or even the entire manuscript, requested a graphical abstract, and - impressed by the professional appearance of the result - did not critically evaluate its scientific focus, originality, and key message. This reaction is understandable. However, it illustrates a central risk: the apparent efficiency of AI can reduce the careful conceptual work that a good graphical abstract actually requires.

A further issue is that AI-generated figures may create an impression of scientific depth without truly providing it. Some submitted abstracts contained unnecessary decorative elements, irrelevant symbolic imagery, or “fantasy graphs” that resembled scientific data visualizations without corresponding to real data. In other cases, systems introduced nonsensical details or obvious visual errors. Such outputs may appear convincing at first glance due to their high visual quality, thereby creating a misleading sense of scientific authority.

Another concern is the increasing similarity of AI-generated scientific graphics. Many figures rely on comparable visual motifs, icons, arrows, glowing molecular structures, or generic biomedical imagery. As a result, originality and individuality may gradually be lost. Scientific communication risks becoming standardized in style while becoming less precise in content.

Importantly, these concerns are not meant as a general criticism of artificial intelligence. AI already provides valuable support in many areas of scientific work, including language editing, image processing, data handling, and graphical design. AI can also assist in preparing graphical abstracts. However, AI-generated output always requires critical human evaluation. Authors remain responsible for deciding whether a figure is scientifically accurate, conceptually focused, and genuinely helpful for readers.

At present, there also appears to be no simple recipe for consistently generating high-quality graphical abstracts using AI alone. Many researchers therefore continue to rely on manual figure preparation, simple graphical software, or hybrid approaches that combine human-designed concepts with selectively used AI-generated elements. In many cases, a simple but conceptually clear figure is far more effective than a visually spectacular but scientifically diluted image.

The increasing availability of AI-generated graphics has also prompted us to reconsider another aspect of editorial policy. Like many journals, we introduced graphical abstracts to improve scientific communication and increase the visibility and accessibility of published articles. In many cases, this goal remains entirely justified. Well-designed graphical abstracts can efficiently communicate key findings and help readers identify articles of particular interest.

However, recent developments suggest that graphical abstracts should not be regarded as universally mandatory. These observations have led us to reflect not only on the quality of individual graphical abstracts, but also on the role we assign to them in editorial policy. Not every scientific study can be meaningfully condensed into a visual format without risking oversimplification, artificial certainty, or conceptual distortion. Particularly concerning is the tendency of graphical abstracts to transform differentiated scientific discussions into visually simplified narratives that may suggest a degree of certainty not fully supported by the underlying data. In some cases, the obligation to provide a graphical abstract may unintentionally encourage the submission of visually elaborate but scientifically weak AI-generated figures created mainly to fulfil a formal requirement.

Interestingly, concerns regarding stylization, oversimplification, and the persuasive visual rhetoric of graphical abstracts had already been raised before the current wave of generative AI tools emerged. In a remarkably foresighted analysis, Sancho Guinda discussed the risk that graphical abstracts may evolve into a form of “disciplinary rhetorical stylisation,” in which visual presentation gradually dominates over conceptual precision and scientific differentiation (Sancho Guinda, 2022[1]). The rapid spread of AI-generated scientific imagery appears to amplify these tendencies.

We have, therefore, come to the conclusion that flexibility may sometimes be preferable to formal uniformity. If authors consider that their work is not well suited for a graphical abstract, we encourage them to briefly explain this decision to the editors in a few sentences. A carefully considered omission may be scientifically more appropriate than the inclusion of a visually attractive but conceptually unconvincing or scientifically oversimplifying figure.

For this reason, we will increasingly pay attention to the scientific quality of graphical abstracts during editorial evaluation. Visual sophistication alone is not sufficient. We strongly prefer figures that communicate a clear scientific message, focus on the essential findings, and reflect genuine intellectual engagement with the presented work. A good graphical abstract should clarify scientific insight rather than merely decorate manuscript content.

## Declaration

### Artificial Intelligence (AI) - assisted technology

No AI tools were used in the preparation of this Editorial.

### Conflict of interest

The authors are editors of EXCLI Journal. They declare no competing financial interests.

## References

[1] Sancho Guinda C (2022). Scientific Stylisation or the “Democracy Dilemma” of Graphical Abstracts. Publications.

